# “We usually see a lot of delay in terms of coming for or seeking care”: an expert consultation on COVID testing and care pathways in low- and middle-income countries

**DOI:** 10.21203/rs.3.rs-3384843/v1

**Published:** 2023-10-04

**Authors:** Gabrielle Bonnet, John Bimba, Chancy Chavula, Harunavamwe N. Chifamba, Titus Divala, Andres G. Lescano, Mohammed Majam, Danjuma Mbo, Auliya A. Suwantika, Marco A. Tovar, Pragya Yadav, Elisabeth L. Corbett, Anna Vassall, Mark Jit

**Affiliations:** London School of Hygiene & Tropical Medicine; Bingham University; Clinton Health Access Initiative; Harare Central Hospital; Kamuzu University of Health Sciences; Cayetano Heredia University; University of the Witwatersrand; Maitama hospital; Padjadjaran University; Socios En Salud Sucursal Perú; National Institute of Virology; London School of Hygiene & Tropical Medicine; London School of Hygiene & Tropical Medicine; London School of Hygiene & Tropical Medicine

**Keywords:** COVID-19, testing, rapid diagnostic tests, self-testing, care pathways, care-seeking

## Abstract

**Background:**

Rapid diagnostic testing may support improved treatment of COVID patients. Understanding COVID testing and care pathways is important for assessing the impact and cost-effectiveness of testing in the real world, yet there is limited information on these pathways in low-and-middle income countries (LMICs). We therefore undertook an expert consultation to better understand testing policies and practices, clinical screening, the profile of patients seeking testing or care, linkage to care after testing, treatment, lessons learnt and expected changes in 2023 in LMICs.

**Methods:**

We organized a qualitative consultation with ten experts from seven LMICs identified through purposive sampling. We conducted structured interviews during six regional consultations, and undertook a thematic analysis of the responses to our questions.

**Results:**

Participants reported that, after initial efforts to scale-up testing (which often encountered delays), the policy priority given to COVID testing has declined. Comorbidities putting patients at heightened risk (e.g., diabetes) mainly relied on self-identification. The decision to test following clinical screening was highly context- and location-specific, often dictated by local epidemiology and test availability. When rapid diagnostic tests were available, public sector healthcare providers tended to rely on them for diagnosis, while private sector providers predominantly used polymerase chain reaction (PCR) tests. Positive test results were generally taken at ‘face value’ by clinicians, although negative tests with a high index of suspicion may be confirmed with PCR. However, even with a positive result, patients were not always linked to care in a timely manner because of reluctance to receiving care or delays in returning to care centres upon clinical deterioration. Countries often lacked multiple components of the range of therapeutics advised in WHO guidelines: notably so for oral antivirals designed for high-risk mild patients. Severely ill patients mostly received corticosteroids and, in higher-resourced settings, tocilizumab.

**Conclusions:**

Testing does not always prompt enhanced care, due to reluctance on the part of patients and limited therapeutic availability within clinical settings. Any analysis of the impact or cost-effectiveness of testing policies post pandemic needs to either consider investment in optimal treatment pathways or constrain estimates of benefits based on actual practice.

## Background

The COVID pandemic has led to a large global disease burden and disruption to economic life since the emergence of SARS-CoV-2 in late 2019. More recently, the disease impact has receded as a result of both natural and vaccine-derived population immunity, leading to the declaration by the World Health Organization (WHO) of the end of the emergency phase of the pandemic on 5 May 2023(1). With the rapidly changing epidemiological situation and development of therapeutics, national COVID policies about pharmaceutical and non-pharmaceutical interventions have been evolving and have increasingly focused on reducing the disease burden in vulnerable people rather than controlling transmission.

Rapid diagnostic tests (RDTs) have played an important role during the emergency phase of the pandemic as they were used to identify infected people and reduce their contacts with others. During that period, guidance and strategies on RDT use were released by countries and institutions including WHO(2) (3), African Centres for Disease Control and Prevention(4), and multiple LMICs(5) (6) (7) (8). Most guidance emphasised support for infection control and economic reopening. Now that transmission prevention is no longer the primary focus, RDTs may still have an important role to play because of their ability to expand testing and facilitate faster linkage to care. Hence, it is important to assess the cost-effectiveness of RDT-supported COVID care pathways. This requires the design of pathways that reflect local practice. WHO has released extensive clinical screening, testing and therapeutic guidelines(9). However, guidance and/or practices in LMICs are likely to differ because of test shortage, differing patient profiles and treatment availability (a course of some therapeutics may cost a thousand dollars(10)). Further, experience with other RDTs has shown that there are often challenges in identifying and reaching out to vulnerable populations and linking to appropriate care(11) (12) (13). We therefore solicited, through this study, LMIC experts’ insights on actual clinical screening, testing, treatment and care practices pertinent to their own country setting, how vulnerable population subgroups were defined and identified in practice, as well as expected future evolutions in their countries. It was also an opportunity for some experts to reflect on lessons learnt from the earlier stages of the pandemic. The results of this study have relevance to cost-effectiveness analyses of RDT use in LMICs.

## Methods

We recruited experts from three world regions (Latin America, Africa and Asia) using purposive sampling. Participants were selected by asking collaborators involved in COVID-19 research in these regions to identify suitable candidates or recommend people who could identify them, prioritizing diversity in regions, country income levels, and past COVID-19 burden. We asked for experts with a good knowledge of COVID testing and care pathways in their countries, preferably at the community level. Initially, 11 experts from eight countries were recruited, but one had to withdraw before the start of the study. Hence, the final sample included ten participants from seven countries (India, Indonesia, Malawi, Nigeria, Peru, South Africa and Zimbabwe). The experts worked for local research institutes, universities, international donors, non-governmental organization partners and/or local hospitals. Five reported having supported their government’s COVID response in an advisory capacity or by contributing to developing guidance (Table 1). We obtained written informed consent from all experts. We then asked them to supply any relevant screening, testing or treatment guidelines/protocols in advance, to inform later discussions on the following topics:

- Testing policies and practices (for both self- and clinician-administered tests);
- Clinical screening practices, the profile of patients seeking testing/care, and any patient groups likely to be missed;
- Linkage to care after testing;
- Treatment/care;
- Expected future changes and lessons learnt.

Discussions used online semi-structured interviews with regional expert groups to account for the practical constraints associated with time zones. There were two meetings with each region over Feb.-Mar. 2023, each taking around an hour and a half, with email follow-up for clarification when needed. A final joint meeting with all regions allowed for presentation of the overall consultation results. The experts also contributed to the drafting of the present paper, of which they are co-authors.

The consultations were recorded and transcribed. The results were then analysed through thematic analysis(14). The themes addressed during the discussion reflected the questions prepared in advance of the consultation with only minimal deviations (i.e., discussion of earlier phases of the pandemic). The full protocol and questions to experts are in Supplement.

## Results

Results are summarised in Table 1 and described below.

### COVID testing policies and practices

Participants reported that COVID was no longer a policy priority in six of the seven represented countries, mostly because of low disease burden and/or public attention. Once RDTs became available, the public sector tended to rely on them (six countries), although PCR was also sometimes used (three countries). Public healthcare providers in two countries faced persisting difficulties accessing tests, leading to a predominance of clinical assessment over RDTs. The private sector, when mentioned, was reported to predominantly use PCR testing.

In discussing the availability and scale-up of PCR, clinician-administered RDT and self-testing in their countries, most experts flagged challenges they face or faced. These include a reluctance to approve self-testing and (initially) rapid-testing in the face of a lack of consistent evidence and messaging at the global level, gaps between policies and community implementation, slow facility accreditation for PCR testing, unaccredited facilities providing inaccurate test results, and treatment capacity and funding sustainability (Box 1). These challenges have reduced the ability to test, or treat when test results came too late to be useful. Country testing and management capacity (which would have solely allowed for isolation and surveillance), sometimes differed from the expectation of patients and even health professionals (who expected better treatment). One participant, however, highlighted that their country was among the first to initiate self-testing worldwide, in May 2021, so testing policies were not uniformly slow to be set in place.

When testing was undertaken, a positive clinician-administered RDT (at least from an accredited facility) was taken as a true positive (at face value) and retesting was not conducted. Negative results, however, may be confirmed, generally with PCR (RDTs may also be used in two countries), particularly if there was high clinical suspicion of COVID-19 or clinical deterioration to severe illness. Four countries still do not have a fully implemented self-testing policy, but respondents reported that self-test kits are often available for direct purchase by patients in some form, either in pharmacies, other stores or online.

#### Box 1: Participants’ feedback about challenges to COVID-19 testing scale-up

Regarding PCR testing expansion: “massification was not achieved partly because of […] this [PCR testing] lab approval and certification system, […] hence for a long while there have been large delays in […] diagnostic tests. [This] has almost completely prevented their use for any clinical decision”.

On RDT policy development: “there has also been […] great resistance to the use of antigen tests because of a lack of international consensus […] and the possibility of false positives”.

Further: “there’s always this gap. [… in which] the testing strategy […] did not really go down fast in the community”. “Generally, some health facilities don’t […] have even the RDTs now to do tests, and some might have [had tests] but […] [they] might be out of stock because most of them are supported by non-governmental organizations. [It is] not really like the government [is] buying the tests now or they are out of stock also”. Meanwhile: “there are other labs that are not accredited […] [and] we don’t usually [consider those results] as serious.”

RDTs sometimes became available without treatment capacity, but people “talked a lot about diagnosing to treat, but there was no way [to do so] and very little was done about diagnosing to isolate”.

Finally, “most of the health workers have reservations [regarding self-testing]. One of the things that they want is to develop a platform where people can report their test [to do surveillance]”.

### Profile of patients seeking testing or care and clinical screening

Participants reported that the most common alternative diagnoses for patients presenting with COVID-like symptoms were influenza (mentioned in four countries), malaria (three countries), tuberculosis or respiratory syncytial virus (two countries each). Participants from two countries highlighted insufficient diagnosis of other infections.

We proposed figures of 55–75% sensitivity and 60–90% specificity for clinical assessment of symptomatic cases as a starting point for discussion. Among participants that fed back on these figures, no major trend (always higher or always lower) was discernible, but the range of plausible values is likely broader (see Table 1). Participants from two countries highlighted the impact of transmission levels in the community on clinical suspicion, while testing and/or clinical capacity constraints were reported to inform the decision to test when suspicion is present. This decision may be very context- and location-specific, influenced by both objective and subjective elements (Box 2).

Finally, the most cited subgroups of patients with a heightened risk of complications from COVID were elderly patients and patients with hypertension or diabetes. Identification of co-morbidities was considered potentially inaccurate, particularly in outpatient settings where assessment usually relies on self-reporting. Following positive results, there was perception of reluctant or delayed presentation to care centres, particularly from the poorest and older adults. Costs to patients of accessing care (direct and opportunity) and fears of acquiring infection or lack of trust in the healthcare system were considered to drive this under-representation. Policies of full isolation of patients and not returning the bodies of patients who had died to families have sometimes heightened the reluctance to present to hospitals.

#### Box 2: Participants’ feedback on clinical screening, testing and patients’ test seeking

On testing decisions: “Even though there is a definition, let us say, operational of COVID suspicion in the technical norms, it ends up at the discretion of the health professional who is at that moment in the testing area […] This has to do with the availability of tests […] sometimes they put caps [on numbers that can be tested]. There has always been some prioritization associated with the probability of improving the person’s prognosis […] which generally meant at the beginning [of the pandemic] the possibility of having a bed”.

Regarding differential diagnoses: “it is not that they were confused, I would rather say that they forgot to continue searching for tuberculosis in a country where it is prevalent”. However, now “the doctors often say […] this is not COVID”.

Too few seek a test: “We usually see a lot of delay in terms of coming for or seeking care”. When finally they present to facilities: “the index of suspicion […] will be very high because some of them might definitely have clear symptoms, you know, most of them.” In another country: “I think it’s quite clear people are not afraid of COVID at the moment and everyone sees it as very mild. Even […] high-risk individuals.”

The situation has sometimes been worse for older patients: “mortality among them has been extremely high […]. They entered health services late hence survival rates from the point of entry was really low […]. Nobody wanted to bring them [to hospitals] out of fear that they would get infected […], and the other thing is that many cases were left at home for them to die with their families given that, if they brought them to hospitals […], they would not see them again. They would maybe be hospitalized then incinerated […] Therefore many preferred to keep their older adult at home […] and take care of them until the end.”

### Linkage to care after testing

We discussed linkage to care (including monitoring systems and patients’ care-seeking behaviours) for patients who self-test and those who have been tested by a clinician, sent home because they had mild illness, then worsened at home. All countries have established rules for monitoring tested patients that are sent home, and four countries have established systems such as helplines, virtual hospitals and telemedicine systems (at least in part of the country) to support patients. However, the capacity to effectively monitor patients often appeared insufficient and patients themselves were sometimes reluctant to seek care when they worsened (Box 3). In two countries, it was reported that positive results may lead to a reduction in the likelihood to seek care at a health centre, while in one other country, the perception was opposite. In another country, the result of the test did not appear to affect care-seeking behaviours, while three countries reported that they did not know.

#### Box 3: Challenges with care-seeking among tested patients with worsening disease

Strong advice was given that any worsening should equate rapid return to facilities after observing a number of [patients] ‘dead on arrival’ following recent positive results.

“My experience is that normally, people come really late when they truly cannot breathe any longer, independently from whether they had a positive or negative COVID test […]. They are more afraid of a positive COVID test because they know they could die. [In our experience,] 60% of people did not accept to go to a hospital”.

### Treatment

Mild, low-risk positive patients were most likely to be prescribed drugs to alleviate symptoms, and may also self-medicate with antibiotics, drugs for symptoms, home remedies and vitamins. There was limited difference in doctors’ treatment for mild-low risk patients that tested positive vs. negative, relating mostly to antibiotic use. Oral antiviral use was still not widespread, hence six of seven countries reported no difference in the treatment of low and high-risk patients.

Severe/critical patients may be given oxygen or mechanical ventilation though availability was still constrained in some cases, particularly in the public sector. Corticosteroids were also given in all surveyed countries (sometimes on a case-by-case basis, or with specialist input for diabetics), while tocilizumab was only mentioned as being used for severe patient treatment in three countries, with one noting limited availability. A test-negative patient with symptoms strongly indicative of COVID would still be treated as a COVID patient in five countries, at least in terms of corticosteroid initiation, while waiting for confirmatory test results.

One of the key possible impacts of testing relates to antibiotic use, which in most countries were often taken by both test-positive and test-negative patients. Overall, participants considered that antibiotics would be used more in test-negative patients, or had no clear answer.

### Reported changes later in 2023, opportunities, challenges and lessons learnt

Two countries expected to get expanded access to COVID antivirals. Meanwhile, self-tests may become more readily available in another country. New initiatives include the development of a pandemic preparedness programme and of a network of centres to test for multiple respiratory infections. One participant also expressed the wish to monitor long-COVID, while noting the limited capacity for that purpose, and another highlighted the need to develop home management standards, including around the use of corticosteroids, for people who refuse hospitalization.

Some lessons learnt include how development of in-house reagents has lowered costs in one country. Finally, one participant expressed the concern that WHO’s declaration that COVID-19 is no longer a public health emergency may negatively affect costs and licensing approval processes.

## Discussion

The main findings of these expert discussions were to give important insights on the realities of COVID testing and care pathways in LMICs, with relevance to cost-effectiveness analysis and future pandemic preparedness. Experts reported that RDTs were often employed in settings where COVID tests were available, but that the choice of which patients were tested was highly context-specific reflecting local epidemiology, perceived morbidity and/or test availability. Doctors were reported to have ignored other diseases, such as tuberculosis, at the height of COVID, and were sometimes ignoring COVID now that the peak of the pandemic had passed. Similarly to experiences with other rapid tests(11) (12), challenges were reported with suboptimal linkage to care after a positive COVID RDT, with many patients unwilling to promptly attend care centres especially if older or poor. Finally, the range of treatment and care options available after a positive test was more limited than in the WHO guidelines(9).

Evaluation of different COVID testing strategies should be based on understanding of conditions in the local context, considering treatment availability, risk identification, and monitoring systems, rather than assuming testing alone will improve outcomes. Guidelines should address the appropriateness of testing if limited or no treatment is available. Further, like with other diseases(15), testing should not be implemented alone but, to have an impact, it should form part of a comprehensive package of COVID care, with adequate resources to address key constraints including training and availability of treatment.

As with other RDTs(16) (17), patients who self-test do not always report their results or promptly seek care. This is also the case for professionally-tested patients with mild disease whose clinical status deteriorates. Further, a positive test result may sometimes even reduce care-seeking. In-depth qualitative research is needed to understand patient behaviours and their drivers and barriers across different LMIC populations. Optimising willingness to report and act promptly on a positive self-test will increase the likelihood that self-testing will be cost-effective.

Finally, our results illustrate the extent to which efforts to scale-up COVID testing faced and will continue to face major challenges, ranging from a lack of scientific consensus to administrative and funding challenges or healthcare staff reluctance. This highlights the need for advanced preparedness and protocols to enable smooth approval and implementation of diagnostic policies during pandemics.

One limitation of this work is our small sample size (ten respondents from seven countries), although we included respondents from countries in a range of geographical regions, income levels and pandemic severity to ensure maximum variety in participants. Hence, the results should be interpreted as providing general insights rather than precise figures. Furthermore, not all participants had expertise or direct experience of all issues being discussed. While some countries had two participants with complementary profiles to provide better insights, answers may still not always reflect the entire range of contexts (from small rural centres to large hospitals) found within each country. We also cannot exclude some desirability bias, as experts may have sometimes been tempted to report on ideal behaviours instead of actual practices, though the many challenges they flagged suggests this bias may not have been widespread. Finally, this consultation was an opportunity for participants to provide some insights as to historical successes and challenges in their countries. However, most of the discussion focused on the present time, meaning that the practices discussed generally reflect the situation in early 2023 rather than throughout the pandemic.

## Conclusions

Our findings have important implications for future research, in particular cost-effectiveness analyses, and are already being used to inform a decision analytic model comparing different COVID testing options for severe cases in LMICs, given countries’ specific screening and testing practices and treatment capacities. Our results highlight obstacles that could reduce the cost-effectiveness of COVID testing in some LMICs, including insufficient availability of key therapeutics, limitations of relying on self-reporting of risk status and suboptimal care-seeking. Complementary, context-specific research (e.g., on care-seeking behaviours) is important. From a policy perspective, our findings suggest that COVID testing needs to be accompanied by investment in comprehensive packages to ensure testing is associated with optimal care pathways.

## Figures and Tables

**Table 1:** Summary of participants’ contributions

Questions/themes	Numbers
Profile of participants (some have multiple affiliations)	Research institute or university (5), hospital (2), international organization e.g., donor, nongovernmental organization, WHO, etc. (4). Gave support/advice to the ministry of health (5)
**Presenting patient profile and screening**	
Under-represented groups in presenting patients	Older adults (2), labourers (1). Sometimes over-representation of formal workers to get sick leave (1)
Most common high-risk conditions	Old age (7), diabetes (6), hypertension (6), immuno-compromised (2), obesity (2) chronic non-communicable diseases (1), mining work (1), pregnancy (1), sickle cell disease (1)
Comorbidity assessment	Declarative (3), measurement (2) - depends on context
Common alternative diagnoses for COVID-like symptoms	Influenza (4), “respiratory illnesses” or “other respiratory illnesses” (4), malaria (3), respiratory syncytial virus (2), tuberculosis (2), adenovirus (1), Nipah (1), parainfluenza (1), pneumonia (1), rhinovirus (1) and typhoid (1).
Sensitivity and specificity of clinical screening	Is 55–75% sensitivity reasonable? 4 answers: much less, reasonable, higher, 80% sensitivity Is 60–90% specificity reasonable? 3 answers: less, reasonable, higher
**COVID-19 professional and self-testing policies and practices**	
Is COVID-19 testing still a policy priority?	A priority (0), no longer a priority (6), still a priority, alongside testing for other respiratory pathogens (1). Reason: low burden (2), other diseases (1), low demand (1)
Modalities of professional patient testing and assessment	In the public sector: RDT (6), PCR (3), clinical assessment (2); in the private sector (mentioned by 3 countries): PCR (3), RDT (1)
Confirmatory testing practices	Do not confirm positives (7) Re-test/confirm negatives if suspicious case (7), with PCR/real-time PCR (7) or with RDTs (2) Re-test to exit quarantine (1)
Self-testing policy	None (1), not yet approved (2), approved but not implemented (1), implemented (3)
Self-testing availability	Pharmacy (3 + 1 with limited availability), other sources e.g., online (2), 1 country with plan for use within contact tracing
**Monitoring and linkage to care**	
Is monitoring/enhanced monitoring practiced?	In theory, but with caveats (3), yes, at least if high risk (2), yes in the norm (1), unsure (1)
Helpline or telemedicine system	Available in at least some of the country (4 countries)
Self-tester care-seeking if positive vs. untested or negative	Less if positive (2), same (1), more (1), unclear/unsure (3)
How likelier is a worsening patient to present to hospital when positive vs. negative?	Likelier (0), as likely (1), less likely (1), no idea/no answer (5), note: many only come when severe/critical (3 countries)
**Treatment**	
Self-medication	Antibiotics (5), symptomatic drugs/home remedies/vitamins (5), ivermectin (2)
Prescriptions for mild low-risk positive cases	Symptomatic drugs/home remedies/vitamins (6), Antibiotics (2), Antivirals (1) Patient demand for ivermectin, at least at some point (4)
Difference in treatment for mild low-risk untested or negative (as compared to positive vases)	Same treatment (1), More use of some medicines (2) e.g., antibiotics, antihistamines or Tamiflu
Additional treatment (compared to low-risk) for mild high-risk patients who test positive	No official added treatment (6), antivirals, as part of the official treatment (1) or asked by some patients (1), anticoagulants (1)
Treatment for severe/critical positive patients	Oxygen/ventilation (7): availability may vary from home oxygen to limited supply, corticosteroids (7) sometimes with specialist input (diabetics) or on a case-by-case basis, antibiotics when needed (3), Tocilizumab (3), not always systematically available, antivirals (1 + 1 country unclear), anticoagulants based on doctor evaluation (1), convalescent plasma at some point (1)
Difference in treatment for severe/critical negative patients as compared to positive patients	No COVID assumption (1), COVID assumption (until confirmation) initiation of steroids (5) - may depend on level of suspicion, antibiotics (1), heparin (1)
Antibiotic use in positive vs. negative patients	Higher use in negative patients (4), with one country highlighting that use would be the same for self-testers, but lower for provider administered testing; ambiguous answer (1); no answer (2). Quantitative answers: two countries fed back on proposed figures: 10–50% antibiotic use in positives seemed correct, 30–75% use in negatives seemed either correct or too high, and a 10–50% difference in antibiotic use between positives and negatives seemed correct for both countries.
**Evolutions, lessons, opportunities and challenges**	
Expected changes in 2023	Improved access to antivirals (2) and self-tests (1), pandemic preparedness programme (1), network of centers to test for multiple respiratory infections (1), change in surveillance: more wastewater and genomic surveillance (1)
Lessons, opportunities and challenges	- At the beginning, costs were high, but efforts to make in-house reagents have helped lower the costs. - We should be monitoring “long-COVID”, but capacity to follow-up on these patients is lacking. - Once the emergency officially ends and government supplies run out, things will evolve in terms of the costs and licensing approval process, which takes longer than with emergency authorization. - It would be useful to develop home management standards for severe patients who refuse hospitalization.
Profile of participants (some have multiple affiliations)	Research institute or university (5), hospital (2), international organization e.g., donor, NGO, WHO, etc. (4). Gave support/advice to MoH (5)
**Presenting patient profile and screening**	
Under-represented groups in presenting patients	Older adults (2), labourers (1). Sometimes over-representation of formal workers to get sick leave (1)
Most common high-risk conditions	Old age (7), diabetes (6), hypertension (6), immuno-compromised (2), obesity (2), chronic NCDs (1), mining work (1), pregnancy (1), sickle cell disease (1)
Comorbidity assessment	Declarative (3), measurement (2) - depends on context
Common alternative diagnoses for COVID-like symptoms	Influenza (4), “respiratory illnesses” or “other respiratory illnesses” (4), malaria (3), RSV (2), TB (2), adenovirus (1), Nipah (1), parainfluenza (1), pneumonia (1), rhinovirus (1) and typhoid (1).
Sensitivity and specificity of clinical screening	Is 55–75% sensitivity reasonable? 4 answers: much less, reasonable, higher, 80% sensitivity Is 60–90% specificity reasonable? 3 answers: less, reasonable, higher
**COVID-19 professional and self-testing policies and practices**	
Is COVID-19 testing still a policy priority?	A priority (0), no longer a priority (6), still a priority, alongside testing for other respiratory pathogens (1). Reason: low burden (2), other diseases (1), low demand (1)
Modalities of professional patient testing and assessment	In the public sector: RDT (6), PCR (3), clinical assessment (2); in the private sector (mentioned by 3 countries): PCR (3), RDT (1)
Confirmatory testing practices	Do not confirm positives (7) Re-test/confirm negatives if suspicious case (7), with PCR/real-time PCR (7) or with RDTs (2) Re-test to exit quarantine (1)
Self-testing policy	None (1), not yet approved (2), approved but not implemented (1), implemented (3)
Self-testing availability	Pharmacy (3 + 1 with limited availability), other sources e.g., online (2), 1 country with plan for use within contact tracing
**Monitoring and linkage to care**	
Is monitoring/enhanced monitoring practiced?	In theory, but with caveats (3), yes, at least if high risk (2), yes in the norm (1), unsure (1)
Helpline or telemedicine system	Available in at least some of the country (4 countries)
Self-tester care-seeking if positive vs. untested or negative	Less if positive (2), same (1), more (1), unclear/unsure (3)
How likelier is a worsening patient to present to hospital when positive vs. negative?	Likelier (0), as likely (1), less likely (1), no idea/no answer (5), note: many only come when severe/critical (3 countries)
**Treatment**	
Self-medication	Antibiotics (5), symptomatic drugs/home remedies/vitamins (5), ivermectin (2)
Prescriptions for mild low-risk positive cases	Symptomatic drugs/home remedies/vitamins (6), Antibiotics (2), Antivirals (1) Patient demand for ivermectin, at least at some point (4)
Difference in treatment for mild low-risk untested or negative (as compared to positive vases)	Same treatment (1), More use of some medicines (2) e.g., antibiotics, antihistamines or Tamiflu
Additional treatment (compared to low-risk) for mild high-risk patients who test positive	No official added treatment (6), antivirals, as part of the official treatment (1) or asked by some patients (1), anticoagulants (1)
Treatment for severe/critical positive patients	Oxygen/ventilation (7): availability may vary from home oxygen to limited supply, corticosteroids (7) sometimes with specialist input (diabetics) or on a case-by-case basis, antibiotics when needed (3), Tocilizumab (3), not always systematically available, antivirals (1 + 1 country unclear), anticoagulants based on doctor evaluation (1), convalescent plasma at some point (1)
Difference in treatment for severe/critical negative patients as compared to positive patients	No COVID assumption (1), COVID assumption (until confirmation) initiation of steroids (5) - may depend on level of suspicion, antibiotics (1), heparin (1)
Antibiotic use in positive vs. negative patients	Higher use in negative patients (4), with one country highlighting that use would be the same for self-testers, but lower for provider administered testing; ambiguous answer (1); no answer (2). Quantitative answers: two countries fed back on proposed figures: 10–50% antibiotic use in positives seemed correct, 30–75% use in negatives seemed either correct or too high, and a 10–50% difference in antibiotic use between positives and negatives seemed correct for both countries.
**Evolutions, lessons, opportunities and challenges**	
Expected changes in 2023	Improved access to antivirals (2) and self-tests (1), pandemic preparedness programme (1), network of centers to test for multiple respiratory infections (1), change in surveillance: more wastewater and genomic surveillance (1)
Lessons, opportunities and challenges	- At the beginning, costs were high, but efforts to make in-house reagents have helped lower the costs. - We should be monitoring “long-COVID”, but capacity to follow-up on these patients is lacking. - Once the emergency officially ends and government supplies run out, things will evolve in terms of the costs and licensing approval process, which takes longer than with emergency authorization. - It would be useful to develop home management standards for severe patients who refuse hospitalization.

## Data Availability

All data generated or analysed during this study are included in this published article.

## Abbreviations

COVID-19: coronavirus disease
LMICs: low-and-middle-income countries
PCR: polymerase chain reaction
RDT: rapid diagnostic test
SARS-CoV-2: severe acute respiratory syndrome coronavirus 2
WHO: World Health Organization
